# ACE2 and LOX Enzyme Inhibitions of Different Lavender Essential Oils and Major Components Linalool and Camphor

**DOI:** 10.1021/acsomega.2c04518

**Published:** 2022-10-05

**Authors:** Sevde Nur Biltekin, Ayşe Esra Karadaǧ, Betül Demirci, Fatih Demirci

**Affiliations:** † Department of Pharmaceutical Microbiology, School of Pharmacy, Istanbul Medipol University, Beykoz, Istanbul 34810, Turkey; ‡ Institute of Sciences, Istanbul University, Istanbul 34116, Turkey; § Department of Pharmacognosy, School of Pharmacy, Istanbul Medipol University, Beykoz, Istanbul 34810, Turkey; ∥ Graduate School of Health Sciences, Anadolu University, Eskişehir 26470, Turkey; ⊥ Department of Pharmacognosy, Faculty of Pharmacy, Anadolu University, Eskişehir 26470, Turkey; # Faculty of Pharmacy, Eastern Mediterranean University, N. Cyprus, Mersin 10, Famagusta 99450, Turkey

## Abstract

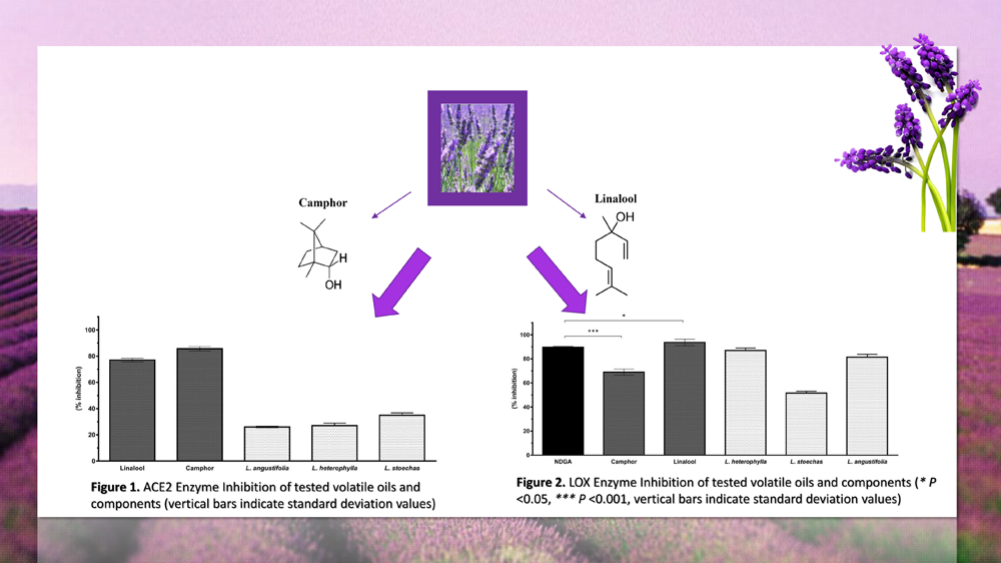

In this present study, *Lavandula angustifolia*, *Lavandula stoechas*, and *Lavandula × heterophylla* essential oils and their main compounds linalool and camphor were evaluated *in vitro* for lipoxygenase enzyme (LOX) and for angiotensin converting enzyme 2 (ACE2) inhibition potential. The chemical compositions of *L. angustifolia*, *L. stoechas*, and *L. heterophylla* essential oils were confirmed both by gas chromatography–mass spectrometry and gas chromatography–flame ionization detection, where 22.4, 0.9, and 30.6% linalool and 17.8, 54.7, and 15% camphor were identified for each oil among other components, respectively. Enzyme inhibitory activity studies were performed at 20 μg/mL for the tested essential oils, whereas for linalool and camphor concentrations, 5 μg/mL was used. The ACE2 inhibitions of *L. angustifolia*, *L. stoechas*, and *L. heterophylla* essential oils were 25.4, 34.1, and 27.1%, while the LOX inhibitions were observed as 79, 49.1, and 86.7%, respectively. In addition, linalool and camphor showed remarkable ACE2 inhibition with 77.1 and 85.1%, whereas the LOX inhibition was observed at 92 and 67.2%, respectively. In conclusion of the initial findings, further detailed *in vivo* studies are needed to confirm the safe use.

## 1 Introduction

Lavender essential oils generally consist of mixtures of mono- and sesquiterpenes and their esters, alcohols, ketones, and oxides along with other volatile compounds. The major components are mostly linalyl acetate, linalool, 1,8-cineole, terpinen-4-ol, β-ocimene, and camphor among others.^1^ Commercial essential oils are mainly produced by distillation from *Lavandula latifolia* Medik., *Lavandula angustifolia* Mill., or *Lavandula* hybrid cultivars. Other species, especially *L. stoechas* L., are recorded to be used ethnobotanically.^2^ However, *L. angustifolia* essential oil and extract are preferred by the cosmetic and food industry due to their low camphor and high linalool contents. Lavandin (*L. x intermedia)* is not the first preference of the industry due to the relatively high camphor content. However hybrid species are used successfully in antifungal, antibacterial, and antiseptic applications.^3−5^

*L. angustifolia* is used for its antimicrobial potential against human pathogenic fungi and bacteria.^6,7^ According to a recent work, *L. angustifolia* essential oil was evaluated *in vitro* against the H1N1 virus and showed remarkable antiviral effects.^8^ Also, *L. x heterophylla* Viv. (syn. *L. x hybrida*) showed strong antibacterial effects against human pathogenic strains.^4^ In addition, *L. stoechas* essential oil is more known with its antifungal effect.^9,10^ Due to its anti-inflammatory properties, *L. angustifolia* essential oil was studied for pathologies and associated diseases.^11^ The major component linalool showed also *in vivo* inhibition of inflammatory mediator production.^12^*Lavandula* species are known and used for their anxiolytic potential, and *L. angustifolia* essential oil medications are available in pharmaceutical form.^13^

In this present study, the potential biological effects of different *Lavandula* essential oils and their major compounds, namely, linalool and camphor, were evaluated by *in vitro* ACE2 and LOX enzyme assays comparatively. To the best of our knowledge, this study is the first work that uses *L. angustifolia*, *L. stoechas*, and *L. heterophylla* essential oils and linalool and camphor for their *in vitro* LOX and ACE2 enzyme inhibitory potential.

## 2 Results and Discussion

### 2.1 GC/MS and GC-FID Analyses

The essential oil compositions (as relative percentages, %) of the tested *Lavandula sp.* are reported in Table 1 with the details up to 97%. When compared, *L. angustifolia* and *L. heterophylla* essential oils showed a relatively high linalool percentage, while camphor percentage was found in high relative amounts in *L. stoechas* essential oil as shown also in the table. The major components of *L. angustifolia* essential oil were identified as 22.4% linalool, 19.2% linalyl acetate, 17.9% camphor, 12.3%1,8-cineole, and 3.8% borneol. The major components of *L. stoechas* oil were characterized and confirmed as 54.7% camphor, 19.2% α-fenchone, 5.4% bornyl acetate, 2.5% 1,8-cineole, and 2.5% camphene. The analyses showed that *L. × heterophylla* essential oil major volatile components were 30.6% linalool, 19.6% linalyl acetate, 15% camphor, 11.3% 1,8-cineole, and 4.2% borneol. According to the analytical results, the linalool content was found relatively high in *L. angustifolia* and *L. heterophylla* oils, while the camphor content was found high in *L. stoechas* essential oil. It was observed that the *L. angustifolia* and *L. heterophylla* oils are in compliance with the European Pharmacopeia in terms of their linalyl acetate and linalool contents. The essential oils analyzed in terms of linalyl acetate, linalool, camphor, borneol, and 1,8-cineol, which are among the important metabolites of *Lavandula* species, were also found to be rich.^1,14^

**Table 1:** Chemical Composition (%) Data of the Tested *Lavandula* Essential Oils

RRI	compound	*L. angustifolia*	*L. heterophylla*	*L. stoechas*
1014	tricyclene			0.3
1032	α-pinene	0.3	0.3	1.3
1076	camphene	0.7	0.5	2.5
1118	β-pinene	0.2	0.4	0.1
1132	sabinene		0.1	
1174	myrcene	0.2	0.2	
1203	limonene	0.4	0.7	0.4
1213	1,8-cineole	12.3	11.3	2.5
1246	(*Z*)-β-ocimene	tr	0.4	
1266	(*E*)-β-ocimene	tr	1.3	
1271	3-octanone		tr	
1280	*p*-cymene	0.4	0.3	0.5
1290	terpiolene		0.1	
1353	hexyl isobutyrate	0.1		
1386	octenyl acetate	0.4	0.2	
1406	α-fenchone			19.2
1424	hexyl butyrate	0.2	0.1	
1450	*trans*-linalool oxide (furanoid)	3.0		0.7
1474	camphenilone			0.5
1478	*cis*-linalool oxide (furanoid)	2.5		0.5
1532	camphor	17.9	15.0	54.7
1553	linalool	22.4	30.6	0.9
1565	linalyl acetate	19.2	19.6	0.6
1583	α-santalene	0.7	0.8	
1591	bornyl acetate	0.2	0.3	5.4
1611	terpinen-4-ol		0.8	
1612	β-caryophyllene	1.4	2.2	
1617	lavandulyl acetate	1.9	1.8	
1616	hotrienol	1.6	0.8	0.1
1648	myrtenal			0.5
1662	pulegone			0.1
1670	*trans*-pinocarveol			0.5
1683	*trans*-verbenol			0.5
1686	lavandulol	1.4	1.7	
1690	lavender lactone	0.2		
1704	myrtenyl acetate			0.1
1706	α-terpineol	1.0	1.2	
1719	borneol	3.8	3.0	1.5
1725	verbenone			1.4
1733	neryl acetate	0.2		
1750	*cis*-linalool oxide (pyranoid)	0.3		
1751	carvone			0.3
1765	geranyl acetate	0.4	0.4	
1770	*trans*-linalool oxide (pyranoid)	0.3		
1786	*ar*-curcumene	0.3		
1804	myrtenol	tr		0.3
1808	nerol	tr	1.4	
1845	*trans*-carveol			0.3
1857	geraniol	0.2	0.3	
1864	*p*-cymen-8-ol	0.3	0.3	1.0
1961	3,7-dimethyl-1,5-octadien-3,7-diol	0.3	tr	
2008	caryophyllene oxide	0.9	0.6	
2104	viridiflorol	0.1	tr	
2238	carvacrol		0.1	
2255	α-cadinol			0.4
	total	95.7	93.8	97.3

### 2.2 Enzyme Inhibitory Activity

The *in vitro* LOX enzyme and ACE2 inhibitory activities of the *L. angustifolia*, *L. stoechas*, and *L. × heterophylla* at 20 μg/mL concentration and their major components linalool and camphor at 5 μg/mL concentrations were evaluated.

#### 2.2.1 ACE2 Inhibition

**Figure 1:**
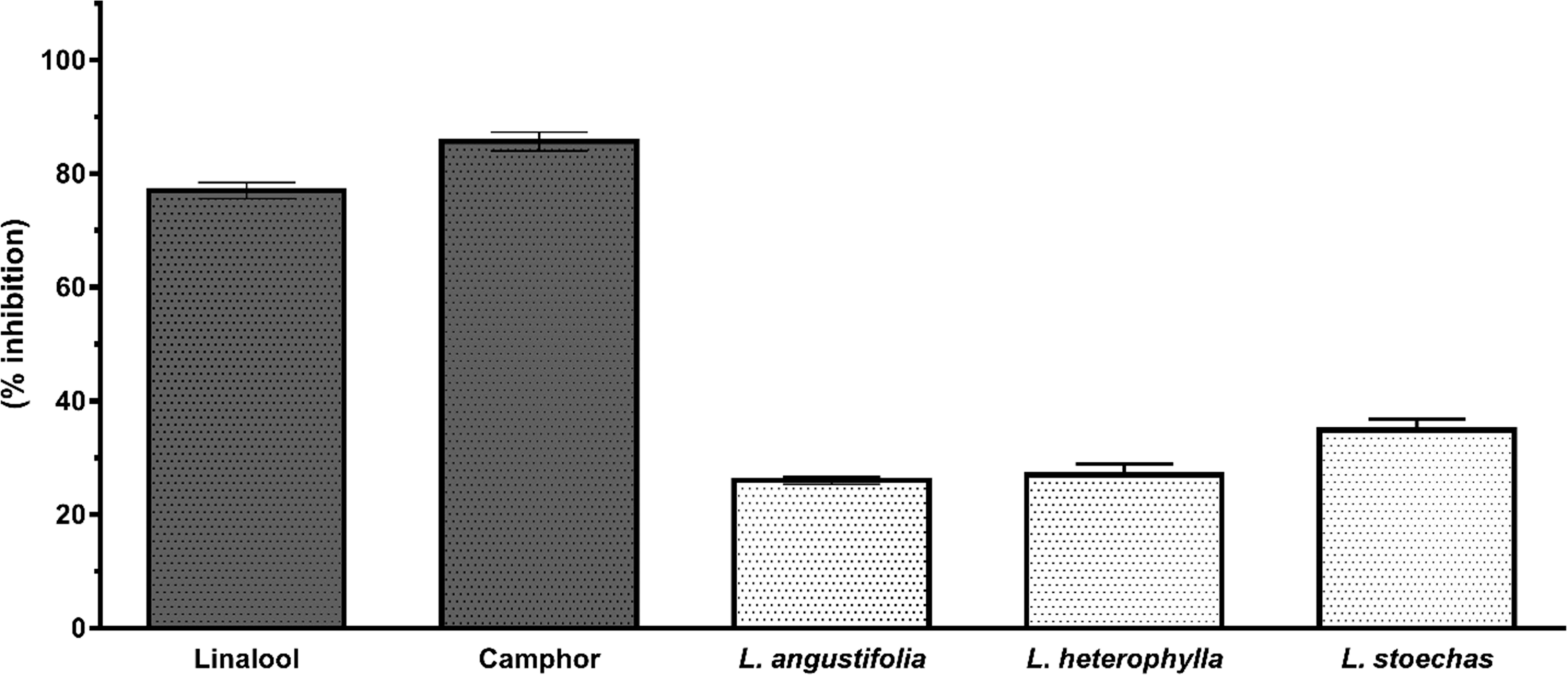
ACE2 enzyme inhibition of tested essential oils and major components (vertical bars indicate standard deviation values).

Camphor showed relatively higher ACE2 inhibitory activity than linalool and the tested *Lavandula* essential oils. *L. angustifolia,L. stoechas,* and *L. heterophylla* essential oils showed 25.4 ± 0.88%, 34.1 ± 0.9%, and 27.1 ± 0.98% ACE2 enzyme inhibitory activity, respectively, as shown in Figure 1. The pure major compounds camphor and linalool showed 85.1 ± 0.95% and 77.1 ± 0.4% ACE2 enzyme inhibitions, respectively.

*Lavandula* essential oils (primarily *L. angustifolia*) were found inhibitory against a broad spectrum of fungi and bacteria.^14−16^ The antimicrobial effect of many essential oils, where linalool was the major component, was remarkable in several studies.^17,18^ Essential oils are also known for their antiviral effects against human herpes viruses, human immunodeficiency virus, influenza virus, and yellow fever virus among others.^19^ Their modes of action are mainly through protease inhibition, genome replication inhibition, or by inhibiting the COVID-19 receptor ACE2 via Pi-H bonding.^8,20^

Essential oils and their main components have lipophilic properties, and some studies show that SARS-CoV-2 virus membrane integrity is disrupted and they have the potential to penetrate the membrane due to such structural features.^20,21^ In a previous study by our group, it was demonstrated that linalool inhibited the ACE2 enzyme also by docking.^22^ Essential oils and their volatile major components also disrupt viral replication, benefiting the host respiratory system through mucus lysis and bronchodilation.^23^ The major compound linalool of *Lavandula* sp. is an important active volatile component present in many essential oils, including *Origanum, Mentha,* and *Laurus* sp. oils, which are also remarkable for antiviral as well as antimicrobial properties. In addition to the broad antimicrobial effects of essential oils, anti-inflammatory and bronchodilator activities are also prominent and well-known.

ACE2, a zinc metallopeptidase, is the only known human homolog of the particular enzyme. It was discovered in early 2000 and is associated mainly with heart function, hypertension, and diabetes. ACE2 is an exopeptidase that catalyzes the conversion of angiotensin 2 to angiotensin 1–7 and l-phenylalanine.^24,25^ ACE2 is a type-I integral membrane glycoprotein that acts as a carboxypeptidase rather than a dipeptidase.^26,27^ The main locations of the receptors of this enzyme, which is active and expressed in most tissues, are cells in contact externally, such as enterocytes of the small intestine and alveolar epithelial cells of the lung.^28^ ACE2 is also found in venous and arterial cells, renal and cardiovascular tissue, and smooth muscle cells.^29^ ACE2 was also one of the receptors for the SARS-CoV, the human respiratory coronavirus NL63, and the novel coronavirus 2019 nCoV/SARS-CoV-2.^30^ Previous studies reported also that ACE2 is one of the essential receptors for various coronavirus types for cell entry.^31,32^ Cardiovascular and lung involvement as complications of coronavirus are the two well-known main causes for death.^30^ Recent studies on essential oils and their active ingredients demonstrated effects on the SARS-CoV virus, such as reducing lethal symptoms, lowering inflammatory responses, inhibiting viral replication, inhibiting viral attachment, and easily penetrating the virus followed by its membrane disruption.^8^ As it was mentioned earlier, ACE2 expression is increased in some regions with the coronavirus. The SARS-CoV-2 virus infects the respiratory epithelium and alveolar macrophages *via* ACE2 receptors in the heart, lungs, and gastrointestinal tract.^33^ Based on the ACE2 enzyme inhibition potentials of essential oils and volatile components, it can be proposed that such substances may be effective in the treatment and prevention of coronavirus cases. In addition, the results of previous antiviral and antimicrobial activity studies with other tested *Lavandula* essential oils, especially *L. angustifolia*, also support the antiviral findings and potential.^3,4,6−8,34^

#### 2.2.2 LOX Inhibition

**Figure 2:**
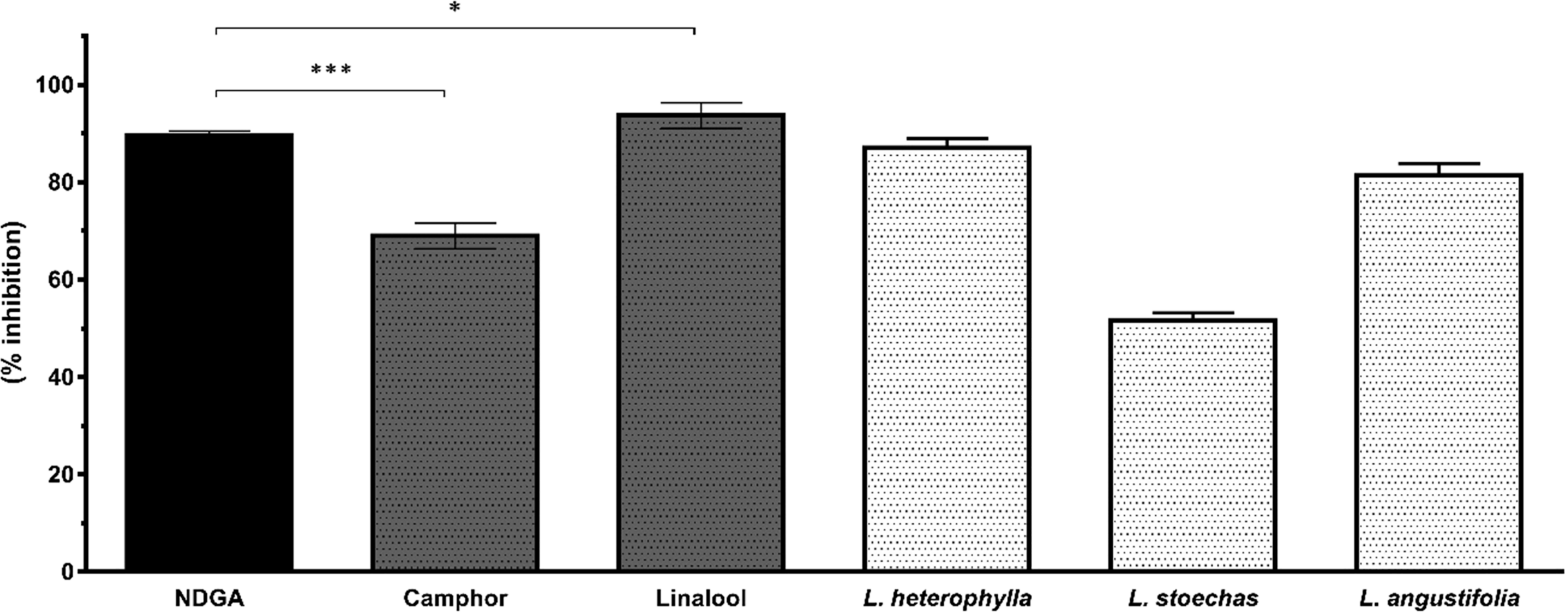
LOX enzyme inhibition of tested essential oils and components (*p < 0.05, ***p < 0.001, vertical bars indicate standard deviation).

The inhibition results of *L. angustifolia*, *L. stoechas*, and *L. × heterophylla* essential oils and linalool and camphor were 79, 49.1, 86.7, 92, and 62.2%, respectively, as illustrated in Figure 2. Also, the anti-inflammatory standard NDGA was compared in the experiments as a positive control, which showed 90.3% LOX enzyme inhibitory activity.

The anti-inflammatory effect of essential oils can be linked to many signal cascade mechanisms, including cytokines, in addition to their antioxidant effects, also thought to be associated with proinflammatory gene expression. It was reported that *L. angustifolia* essential oil showed a significantly higher LOX enzyme inhibition, which may be associated to the combination of linalool and camphor, which are known to have high LOX enzyme inhibition levels.^35^ In another study, TNF-α and IL-6 cytokine levels were measured to test the *in vitro* anti-inflammatory effect of linalool-rich essential oils, and it was claimed that the reason for the significant decrease in cytokine levels may be due to the linalool content along with other components, which is one of the main components.^36^ In another *in vivo* study conducted with a camphor-rich essential oil, a decrease in IL-6 cytokine level was observed.^37^

As it is well-known, the LOX enzyme is effective on the free arachidonic acid (AA) cascade. This enzyme is responsible for proinflammatory leukotrienes (LTs) and leukotriene receptors, which are also increased in expression in many cells such as vascular endothelial and smooth muscle cells related to SARS-CoV-2.^38,39^ It is proposed that LOX inhibitors may be as important as antiviral drugs for modulating SARS-CoV-2 infections as leukotrienes enhance local inflammation in various diseases.^35^

In a previous study, the high LOX inhibition of *Salvia officinalis* was evaluated, and it was stated that this effect may be caused by the high content of camphor.^40^*In vivo* experiments showed recently that linalool has a relatively high anti-inflammatory effect by inhibiting inflammatory mediator production.^12^ Due to its anti-inflammatory properties, *L. angustifolia* essential oil is also traditionally used in aromatherapy.^11^

In this present study, the relatively high LOX inhibition percentages for *L. angustifolia* and *L. heterophylla* essential oils and linalool and camphor, which are the main components, showed potential for anti-inflammatory-related diseases.

As a conclusion of this present study, as *Lavandula* essential oils rich in linalool have a relatively high ACE2 and LOX inhibition, they can be further experimented against coronaviruses using various formulations. Detailed *in vivo* study data for safety and efficacy, however, are needed before antiviral applications.

## 3 Methods

### 3.1 Materials

Linalool, camphor, lipoxygenase enzyme, and other chemicals were acquired from Sigma Aldrich. “Angiotensin II Converting Enzyme (ACE2) Inhibitor Screening Kit” enzyme kit was obtained from BioVision (K310). Commercial *Lavandula* essential oils were supplied by Doallin, İstanbul, Turkey. Voucher samples are deposited at the IMEF Herbarium (herbarium no.: IMEF 1188-1189-1190).

### 3.2 GC-FID and GC/MS Analysis

For gas chromatography–flame ionization detection (GC-FID) analyses, the FID was used at 300 °C (Agilent 6890N GC system). Simultaneous automatic injection was carried out using the same conditions in two identical columns in the gas chromatography–mass spectrometry (GC/MS) system (Agilent 5975 GC-MSD). Relative percentages (%) of the volatiles were calculated using the FID chromatograms. This process was performed by GC/MS Library, MassFinder 3 Library, and in-house “Baser Library of Essential Oil Constituents” by analyzing either authentic samples or the relative retention index (RRI) of *n*-alkanes.^41,42^

### 3.3 Enzyme Inhibitory Activity

#### 3.3.1 ACE2 Inhibition

The standard instructions of “Angiotensin II Converting Enzyme (ACE2) Inhibitor Screening Kit” (BioVision, catalog number: K310) were applied. Stock solutions of the test substances were prepared using DMSO (1%, v/v) and 20 μg/mL each oil; the pure compounds (5 μg/mL) were transferred to the well. The prepared ACE2 enzyme solution was added to all wells except the blank. The substrate solution was prepared and added as 40 μL to each well. The reaction mixture was measured with Ex/Em = 320/420 nm wavelength using a microplate reader (SpectraMax i3) at fluorescence mode after the incubation period. The results were reported as % inhibition values, which were obtained for all samples resulting from triplicate analyses.^42^

#### 3.3.2 LOX Inhibition

The results were measured by the common colorimetric method^43^ and as previously reported.^22^ The % inhibition was calculated as the absorbance change for the minute of enzyme activity compared to absorbance change for a minute of the tested oils and compounds. Nordihydroguaiaretic acid was also used as a positive control. The analyses were performed in duplicate, and results are given as mean and standard deviation (SD).

### 3.4 Statistical Analysis

The statistical analysis was carried out using the GraphPad Prism, version 7.02 (La Jolla, California, USA). *In vitro* data was expressed as mean ± standard deviation (mean ± SD). The *p* < 0.05 was accepted as statistically significant.
